# Innovative Strategies to Reduce Exposure and Expression of the Major Cat Allergen Fel d 1

**DOI:** 10.1002/clt2.70098

**Published:** 2025-09-09

**Authors:** Simone Colosimo, Cristiana Indolfi, Vittoria Frattolillo, Gianluca Mondillo, Alessandra Perrotta, Mariapia Masino, Fabio Decimo, Michele Miraglia del Giudice

**Affiliations:** ^1^ Department of Woman, Child and of General and Specialized Surgery AOU University of Campania “Luigi Vanvitelli” Naples Italy

**Keywords:** cat allergy, dietary interventions, Fel d 1, IgY antibodies, immunotherapy

## Abstract

**Background:**

Fel d 1, the primary allergen produced by cats, is a glycoprotein found mainly in their salivary and sebaceous glands. Due to its small size and stability, it easily becomes airborne and adheres to surfaces, posing a persistent problem for allergic individuals.

**Methods:**

This article reviews innovative strategies aimed at reducing Fel d 1 expression and exposure and mitigating its allergic effects on humans.

**Results:**

A key approach involves dietary supplementation with chicken egg‐derived IgY antibodies specific to Fel d 1. These antibodies neutralize the allergen in the saliva, significantly reducing its transfer to the fur and environmental presence, with clinical studies showing a notable decrease in nasal symptoms among allergic individuals. Additional dietary factors, such as omega‐3 fatty acids, polyphenols, and low‐glycemic index foods, may further modulate allergen production via hormonal and sebaceous pathways. Immunotherapy options include the Fel‐CuMV vaccine for cats and human‐targeted treatments with monoclonal antibodies like REGN1908‐1909, both of which demonstrate significant reductions in allergic symptoms. Genetic modification and hormonal manipulation (e.g., neutering) offer further avenues to lower Fel d 1 expression. Environmental strategies, including HEPA filters and protease treatments, can also help reduce allergen load in households.

**Conclusions:**

Together, these approaches form a multifaceted framework for managing cat allergies, potentially allowing allergic individuals to coexist with their pets. Future research should aim to optimize these interventions and explore synergistic combinations to achieve more effective and personalized allergy management.

**Key Message:**

This review summarizes innovative methods for reducing Fel d 1 production in cats, including genetic, immunological and dietary approaches, with potential implications for allergy prevention in humans.

## 1 Introduction

Fel d 1, a glycoprotein primarily produced in the salivary and sebaceous glands of cats, is the main allergen responsible for feline‐induced allergic reactions in humans [1]. Its biological function is not yet fully understood, but some studies have identified homologous proteins to Fel d 1 in other species of the Felidae family, such as lions, tigers, and cheetahs, suggesting an evolutionary conservation of this protein among felines [2, 3]. During the grooming process, this protein is transferred from saliva to the fur and mixes with sebum, which promotes its persistence; it is then dispersed into the environment through shed fur and dander [4].

Fel d 1 particles are extremely small (generally less than 5 μm in diameter) and lightweight; as a result, they can remain suspended in the air for long periods, adhere to surfaces such as fabrics and carpets, making it difficult to completely eliminate the allergen from homes and very easy for it to be transported from home to workplaces [5]. Cat allergies can cause a variety of symptoms, including allergic rhinitis, conjunctivitis, and asthma [6, 7]. Recent evidence indicates that in children, molecular sensitization to cat allergens—particularly the uteroglobin Fel d 1 and other furry animal lipocalins—is strongly associated with type 2 airway inflammation, blood eosinophilia, and a higher prevalence of asthma‐like symptoms, even in the absence of marked lung function abnormalities [8]. Managing these allergies often involves the use of symptomatic medications, such as antihistamines and corticosteroids, as well as strategies to reduce Fel d 1 expression, including dietary interventions, immunotherapy, genetic modifications, and environmental approaches. This article explores these strategies by integrating findings from numerous recent studies.

## 2 Discussion

### 2.1 Dietary Interventions: Reducing Fel d 1 Through IgY‐Enriched Nutrition

One of the most promising approaches to reducing Fel d 1 relies on the use of IgY antibodies specific to Fel d 1, derived from chicken eggs. Incorporated into cat food, these antibodies neutralize Fel d 1 in saliva, reducing the amount transferred to the fur during grooming [9].

IgY antibodies are avian immunoglobulins that functionally correspond to IgG in mammals. Unlike IgG, IgY does not activate the complement system and does not interact with Fc receptors, thereby reducing the risk of adverse reactions and false positives in immunological assays. This makes them particularly useful in diagnostic and therapeutic applications where such interactions could cause complications [10]. IgY antibodies are produced in large quantities by hens and can be easily isolated from egg yolks, making them a cost‐effective and abundant source of antibodies [9, 10]. A single hen can produce up to 100–150 mg of IgY per egg, with an annual production of approximately 28–42 g of IgY per hen. This makes IgY an economical and abundant antibody source, reducing costs and ethical concerns associated with antibody production in mammals [10].

IgY antibodies offer several advantages over mammalian IgG, including greater stability across different pH levels and temperatures. IgY remains stable within a pH range of 4–11 and can withstand temperatures up to 65°C without losing activity [10].

Several studies have explored the effectiveness of IgY antibodies in reducing Fel d 1 levels in cats, with promising results.

Satyaraj et al. recruited 11 participants allergic to Fel d 1 and placed them in chambers containing blankets used by cats fed a test diet containing anti‐Fel d 1 IgY or a control diet. The exposure showed a significant reduction in total nasal symptoms in subjects exposed to blankets from cats fed the test diet compared to the control diet. The results indicate that lowering active Fel d 1 levels in cats can effectively reduce allergic symptoms in humans [11].

The same author, in a subsequent study, fed 105 domestic shorthair cats a diet containing anti‐Fel d 1 IgY for 12 weeks, ensuring that the diet did not negatively impact their health. During this period, active Fel d 1 (aFel d 1) levels in the cats' fur were measured using ELISA. The study results showed a significant reduction in Fel d 1: in particular, from the third week of treatment, a statistically significant reduction in mean levels of aFel d1 on cat hair compared to baseline was observed (*p* < 0.05), with an average decrease of 47% by the 10th week of treatment. Cats with initially higher aFel d 1 levels (average media 533,3 ± 330,0 μg/g) exhibited the greatest reductions (*p* < 0.001), suggesting that the effectiveness of IgY treatment is particularly pronounced in cats that produce high amounts of the allergen [12].

To determine whether the reduction in allergen levels in cats could translate into decreased symptoms in allergic individuals, researchers conducted controlled exposure studies using IgY dietary interventions.

The use of IgY to reduce the Fel d 1 allergen in cats represents an innovative and promising approach for managing cat allergies in humans. The oral administration of IgY through diet can significantly lower allergen levels in cats without adverse effects on the animals' health. This approach could offer a solution for individuals who, despite being allergic, live with cats, allowing them to spend time with their pets while preserving their health [12, 13].

However, further research is needed to validate these findings and optimize treatment protocols. For instance, it would be beneficial to investigate the optimal IgY dosage required to achieve maximum Fel d 1 reduction and to assess whether significant differences exist among various cat breeds or environmental conditions that may influence treatment efficacy.

Although studies conducted thus far have not identified significant adverse effects, it is crucial to continue closely monitoring the health of cats undergoing treatment, paying particular attention to potential adverse reactions or changes in overall health status. This approach is essential to ensure the long‐term safety and effectiveness of the treatment [12, 13].

Satyaraj et al. [14] also demonstrated that a diet containing anti‐Fel d 1 IgY resulted in a 29.6% reduction in salivary Fel d 1 levels in just 6 weeks (*p* = 0.023), with 81.8% of treated cats experiencing at least a 20% reduction. Moreover, the cats maintained unchanged clinical, biochemical, and behavioral parameters throughout the treatment [15]. Finally, the reduction in Fel d 1 levels translates into a lower allergenic load in the environment, as demonstrated by Wedner et al. [13]: the Total Nasal Symptom Score (TNSS) was significantly lower when participants were exposed to blankets from test‐fed cats compared to the priming exposure (*p* = 0.035), nasal congestion showed a significant reduction in the test group compared to priming (*p* = 0.0055), while itchy eyes and scratchy eyes also improved significantly with *p* = 0.0072 and *p* = 0.015, respectively. No statistically significant differences were observed in the Total Ocular Symptom Score (TOSS) (*p* = 0.72) [12].

In another study, Satyaraj et al. [16] utilized polyclonal antibodies and IgY antibodies derived from eggs of hens immunized with Fel d 1, capable of neutralizing the allergen directly at its source. The antibodies were tested in vitro using cat saliva, a natural source of Fel d 1, and human plasma containing IgE specific to Fel d 1. After incubating the saliva with the antibodies, two main types of experiments were conducted.

In the ELISA test, the binding of Fel d 1 to IgE in human plasma was measured: polyclonal antibodies significantly reduced this binding at dilutions up to 1:2000 (*p* < 0.001), while IgY‐derived antibodies showed a similar effect, with a significant reduction compared to controls (*p* < 0.001).

To date, no data are available in the literature regarding the dissociation constant (Kd) between antibodies and the Fel d 1 allergen. The Kd reflects the strength of binding between the antibody and the allergen. Understanding this parameter is useful in assessing the efficacy of antibody‐based treatments and designing better therapeutics. However, its measurement is technically challenging due to the heterogeneous nature of antibody‐epitope interactions.

In the humanized basophil degranulation model, the saliva treated with antibodies was tested on cells sensitized with Fel d 1‐specific IgE. Degranulation, measured by β‐hexosaminidase release, was reduced by up to 60% at 1:200 dilutions for polyclonal antibodies and up to 40% for IgY (*p* < 0.05). Monoclonal and non‐specific antibodies, however, did not show significant effects.

This study provides a promising basis for the use of blocking antibodies as a safe and effective strategy to reduce allergen exposure in individuals allergic to cats.

Recent evidence further confirms the safety and efficacy of anti‐Fel d 1 IgY‐enriched diets. Hedrick et al. (2024) conducted a blinded, controlled trial evaluating the impact of this diet on kitten growth and health parameters. Their findings demonstrated no adverse effects on growth rate, food consumption, or overall health status, reinforcing the safety of long‐term dietary administration of anti‐Fel d 1 IgY in felines [17].

Complementing these findings, Bousquet et al. (2024) employed the MASK‐air mobile app to conduct a proof‐of‐concept clinical study assessing allergic symptom severity in cat‐allergic individuals exposed to cats fed an IgY‐enriched diet. The study revealed significant improvements in global allergy symptoms, decreased medication use, and better work productivity outcomes. This real‐world clinical evidence substantiates the therapeutic potential of dietary IgY interventions in reducing Fel d 1 allergenicity and improving the quality of life for allergic individuals [18].

Despite the clinical efficacy observed in allergic patients exposed to cats fed with anti‐Fel d 1 IgY‐enriched diets, some methodological limitations must be considered. In particular, the small sample size in some studies may limit the generalizability of the results, and the open‐label design adopted in certain protocols may introduce potential observation bias. Moreover, the involvement of commercial sponsors, such as manufacturers of the IgY‐supplemented diets, could represent a conflict of interest. These considerations do not diminish the value of the available evidence but underscore the need for large‐scale, independent, randomized controlled trials to robustly confirm the long‐term efficacy and safety of such dietary interventions.

### 2.2 Other Dietary Interventions

Diet composition can influence the allergenicity of cats by regulating sebum production, the primary vehicle for Fel d 1 [19]. Androgens, for example, significantly affect sebaceous gland activity; dietary compounds such as polyphenols (found in soy and tomatoes) can inhibit the activity of the enzyme 5α‐reductase, reducing the synthesis of testosterone and dihydrotestosterone. This, in turn, leads to a decrease in sebum and Fel d 1 production.

The ratio between Omega‐6 and Omega‐3 fatty acids in the diet also plays a crucial role: an imbalance favoring Omega‐6 stimulates sebaceous secretion, whereas an increase in Omega‐3 may help reduce inflammation and lower allergen production.

Finally, it has been hypothesized that low‐glycemic index diets, due to their ability to lower insulin and IGF‐1 levels, may decrease sebocyte proliferation and differentiation, further reducing Fel d 1 release.

Although these strategies do not represent a cure for cat allergies, the integration of specific nutrients could serve as a practical method to lower environmental Fel d 1 concentrations, improving allergy symptom management in sensitive individuals [19].

### 2.3 Immunotherapy and Monoclonal Antibodies

Immunotherapy represents an effective approach to desensitizing the human immune system to cat allergens. Among innovative methods, the Fel‐CuMV vaccine (HypoCat) stands out for its ability to induce the production of neutralizing antibodies in cats themselves. In a study conducted by Thoms et al. [20], cats vaccinated with Fel‐CuMV showed a significant increase in IgG antibodies against Fel d 1 (*p* < 0.001), which reduced active allergen levels. This reduction translated into an improvement in allergic symptoms among cat owners and increased tolerance to physical contact with the animals, as assessed through questionnaires [20].

J. Grundström et al. [21] hypothesized that the covalent binding of vitamin D3 (VD3) to the Fel d 1 allergen could enhance the therapeutic effects of specific immunotherapy. Using mice sensitized to Fel d 1 and randomizing them into two groups—one treated with Fel d 1 bound to VD3 (rFel d 1:VD3) and the other treated with Fel d 1 without VD3—the researchers evaluated results in terms of airway hyperresponsiveness (AHR), cytokine levels, bronchoalveolar lavage (BAL) cell counts, and specific antibody levels. Mice treated with rFel d 1:VD3 showed a significant reduction in airway hyperresponsiveness (*p* < 0.05) and pulmonary inflammation, evidenced by a marked decrease in total cells and eosinophils in BAL (*p* < 0.05). Additionally, IL‐5 levels in BAL were significantly lower in the rFel d 1:VD3 group (*p* < 0.05). This approach could therefore represent a strategy to improve the efficacy and safety of immunotherapy by reducing the required allergen dose and potentially shortening treatment duration, leveraging the immunomodulatory properties of vitamin D.

At the same time, the use of monoclonal antibodies has opened new frontiers. REGN1908‐1909, a combination of two humanized monoclonal antibodies, was designed to bind Fel d 1, preventing its interaction with human IgE [22]. Previous phase 1 data in adults with cat allergy and allergic rhinitis, evaluated through a nasal allergen challenge model, demonstrated that REGN1908 and REGN1909 significantly reduced nasal symptom scores in a dose‐dependent manner, with the highest and most sustained effects observed at 300 and 600 mg total doses [23]. These findings supported the subsequent phase 2 investigation in asthma patients. Specifically, the use of REGN1908/909 has proven effective in preventing early asthmatic responses (EAR) in patients with mild asthma and cat allergy. On days 8, 29, 57, and 85 after treatment, treated patients showed improved respiratory function compared to the placebo group. Specifically, the average improvement in FEV1 was 15.15% in the treated group versus 1.59% in the placebo group on day 8 (difference: 13.56%, *p* < 0.001) and 12.73% versus 0.20% on day 85 (difference: 12.54%, *p* = 0.008). Additionally, the incidence of EAR was reduced, with 50% of treated patients not experiencing an acute attack during exposures on day 85, compared to approximately 80% of patients in the placebo group [24].

During treatment, no significant variations in serum IgE levels were observed; however, the drug appears to prevent mast cell activation, thereby reducing degranulation [25]. This therapy could offer an alternative to traditional specific immunotherapy, which still presents significant limitations, such as prolonged treatment duration and variability in outcomes [26]. Immunotherapy with Fel d 1‐expressing vaccines or mAbs is targeted and systemic, potentially useful for those unable to avoid cats. However, it is still at an early stage compared to house dust mite or pollen immunotherapy.

### 2.4 Genetic and Hormonal Approaches

Genetic modification and the selection of hypoallergenic cats represent complementary strategies to reduce Fel d 1. Bioinformatics analyses by Brackett et al. [27] suggest that Fel d 1 is not essential for cat health, making it a candidate for genetic elimination. Preliminary studies indicate that deletion of the Fel d 1 gene could significantly reduce allergen production without adverse effects on cats [28]. However, the practical applicability of this technique requires further research.

The hormonal influence on Fel d 1 production has been analyzed by Ramadour et al. [29], who found that intact males produce significantly higher levels of Fel d 1 than neutered males and females. Neutering, therefore, represents an effective method to reduce allergen production in males. In particular the authors demonstrated that intact male cats produce significantly higher amounts of Fel d 1 on their fur compared to neutered males (geometric mean: 5.46 vs. 1.28 U/g, *p* < 0.0001) and to females, regardless of reproductive status. The effect of neutering appears clearly relevant in males, while no significant differences were observed between spayed and intact females, or between females and neutered males. Moreover, Fel d 1 distribution analysis revealed that over 70% of intact males fell within the highest quartile of allergen concentration, whereas no intact females were represented in that group. This strongly supports the role of androgens in modulating Fel d 1 production.

However, several confounding factors may affect the interpretation of these results. The study population consisted of European breed cats with wide age variability (mean ∼5–6 years, standard deviation > 5 years), introducing potential heterogeneity linked to age or other uncontrolled variables (e.g., environmental conditions, nutrition, stress, dermatological status). Although the sampling procedure was standardized (axillary site, shaving under anesthesia), plasma hormone levels or endocrine markers were not measured—limiting causal inference.

Finally, as the authors themselves acknowledge, neutering does not eliminate the clinical risk: spayed females and neutered males may still produce sufficient levels of Fel d 1 to trigger allergic symptoms in sensitized individuals, especially in environments where the allergen accumulates (e.g., carpets, bedding, soft toys). Therefore, while neutering is a helpful strategy to reduce allergen exposure in male cats, it does not represent a definitive solution and should be considered part of a broader allergen management plan that may include environmental control and additional interventions.

Genomic technologies offer promising prospects for developing hypoallergenic cats by targeting Fel d 1 expression. Zhang et al. [30] reviewed the application of gene‐editing tools such as CRISPR and selective breeding programs aimed at reducing or eliminating Fel d 1 expression in cats. Although these strategies present exciting opportunities for allergy management, the author highlights important ethical, welfare, and environmental considerations that must be addressed before large‐scale implementation [30].

### 2.5 Environmental Measures and Proteases

Environmental measures play a crucial role in managing cat allergies. Björnsdottir et al. [31] demonstrated that implementing environmental controls, such as using HEPA filters, removing carpets, and regular cleaning, significantly reduces Fel d 1 levels in the home environment. Study participants reported a significant improvement in respiratory symptoms and nasal inspiratory flow.

Two randomized, placebo‐controlled clinical trials conducted in standardized environmental exposure chambers (ALYATEC and Dyson Environmental Exposure Chamber) have provided robust evidence supporting the efficacy of HEPA air purifiers in preventing allergic reactions to Fel d 1 in cat‐allergic individuals. Gherasim et al. [32] showed that the use of the Intense Pure Air XL device equipped with an H13 HEPA filter significantly reduced both early asthmatic responses (29.1% vs. 87.5%; *p* = 0.002) and late responses (16.6% vs. 45.8%; *p* = 0.002), while decreasing airborne Fel d 1 levels from 36.3 to 4.7 ng/m^3^. More recently, a second trial [33] confirmed these findings with the Dyson HEPA Big + Quiet Formaldehyde purifier, reporting no early bronchial response in the active group compared to 53.3% in the placebo group (*p* = 0.02) and a 52.2% reduction in rhinoconjunctivitis symptoms (*p* = 0.03). These results support the use of HEPA purifiers as an effective and clinically relevant environmental intervention for managing cat allergy.

Another possibility is the use of proteases to degrade Fel d 1. Papain and subtilisin, for example, have been shown to degrade over 80% of Fel d 1 within minutes and could be incorporated into products such as shampoos, sprays, and air filters [34, 35]. Wells et al. [34] demonstrate that the proteolytic enzyme subtilisin A is capable of degrading the Fel d 1 allergen applied to artificial textile supports. The enzymatic activity was verified under controlled conditions, using acrylic and cotton strips treated with a solution containing recombinant Fel d 1, to which subtilisin was applied at different concentrations. The efficacy was evaluated using a Fel d 1‐specific ELISA test.

The results show a dose‐dependent reduction of the recoverable allergen, with degradation values increasing with higher enzyme concentrations and longer incubation times. The experiment includes a clear dose‐response curve (e.g., from 0.05% to 1% subtilisin), documenting the effect of concentration and time. In addition, a simulated environmental test was conducted in a nebulization chamber, where the enzyme was sprayed onto a support impregnated with Fel d 1, and the subsequently measured aerosol showed a significant reduction in airborne allergen content.

These data provide initial experimental support for the possibility of reducing Fel d 1 exposure through enzymatic degradation in a controlled environment. However, the document does not include studies conducted in real domestic settings, nor evaluations of clinical effects in allergic individuals.

In another more recent patent [35], Wooster et al. describe proteolytic formulations designed to degrade Fel d 1 using enzymes such as subtilisin, papain, bromelain and ficin. The inventors performed in vitro assays on surfaces coated with recombinant allergen and measured protein reduction by Fel d 1‐specific ELISA. Subtilisin achieved degradation levels above 90%–95%, whereas papain proved less potent yet still demonstrated dose‐dependent cleavage of Fel d 1. Although these findings provide initial experimental support for enzyme‐based environmental interventions, the document offers no data from real‐world household settings nor clinical evaluations. Consequently, the results should be viewed as preliminary, and there remains a clear need for peer‐reviewed studies to confirm safety and effectiveness.

A comparative summary of the discussed strategies is provided in Table 1.

**TABLE 1:** Comparison of innovative strategies aimed at reducing Fel d 1 expression in cats and mitigating allergic symptoms in sensitized individuals.

Strategy	Mechanism	Fel d 1 reduction (%)	Study type	Sample size	Strength of evidence
Anti‐Fel d 1 IgY‐enriched diet	Passive immunoneutralization in saliva	29.6% in saliva; 47% on fur	RCT (cats); open‐label study (humans)	11 cat owners	Moderate (small sample size)
Fel‐CuMV vaccine	Active immunization of the cat against Fel d 1	70%–90% in cat tear fluid	Preclinical in vivo	Not specified	Preliminary (lack of clinical data)
REGN1908–1909 monoclonal antibodies	Direct allergen binding preventing IgE engagement	↓ early asthmatic response; ↓ nasal symptoms	RCT in allergic patients	*N* = 56 allergic patients	High (well‐designed RCT)
Fel d 1 gene editing (CRISPR)	Genetic deletion of Fel d 1 chain 2	Potential Fel d 1 elimination; in vitro data only	In vitro; in silico; gene editing in feline lines	*N* = 6 cats; genetic data	Low (no clinical or real‐world data)
Neutering	Hormone‐mediated suppression of Fel d 1 production	75%–90% comparing intact and neutered males (geometric mean)	Observational study on domestic cats	*N* = 221 cats	Moderate (confounding factors uncontrolled)
Subtilisin A (proteolytic enzyme)	Enzymatic degradation of allergen on surfaces	Up to 95% in vitro; only on artificial surfaces	In vitro; simulated environmental spray test	Not applicable	Low (no real‐world data)
HEPA air purifiers	Filtration of airborne Fel d 1	↓ clinical symptoms and decreased EAR (measured by FEV_1_); significant allergen reduction in exposure chamber	2 RCTs in controlled environmental chamber	*N* = 24 and *N* = 30 allergic patients	Moderate (efficacy shown in simulated conditions)

Abbreviation: EAR, early asthmatic response.

### 2.6 Literature Gaps

Despite the promising results of individual strategies to reduce Fel d 1 expression or exposure, several critical gaps in the current literature must be acknowledged. First, no direct head‐to‐head comparative trials are currently available that evaluate the relative efficacy of dietary, immunological, genetic, or environmental interventions. Most studies assess single interventions in isolation, making it difficult to determine the most effective or synergistic approaches for real‐world application. Future clinical trials should incorporate multi‐arm designs that allow for direct comparison of strategies such as anti‐Fel d 1 IgY‐enriched diets versus immunotherapy or environmental purification systems.

Second, although the Fel‐CuMV vaccine and monoclonal antibodies like REGN1908‐1909 have shown significant short‐term benefits [24], the long‐term safety profile of these immunomodulatory approaches remains insufficiently explored. Current studies are limited to short follow‐up periods and relatively small cohorts, and questions persist regarding potential immunogenicity, loss of efficacy over time, and the impact on feline health or immune tolerance in humans. Moreover, genetic interventions such as CRISPR‐mediated Fel d 1 gene deletion, while theoretically promising [28], are still in the preclinical stage, and their safety, reversibility, and off‐target effects require thorough evaluation.

Third, the heterogeneity in methodologies for quantifying airborne Fel d 1 across studies poses a major limitation. Although cascade impactors and ELISA assays are commonly used [5], the lack of standardized protocols impedes comparison across trials and weakens the reproducibility of findings. Variables such as sampling time, environmental conditions, and allergen extraction procedures differ widely, introducing methodological bias. The development of internationally accepted standards for airborne Fel d 1 measurement would enhance the comparability and reliability of future research efforts.

Addressing these gaps is essential for the development of robust, integrated approaches to managing cat allergy.

## 3 Conclusions

Dietary approaches, immunotherapy, and genetic interventions offer promising solutions for managing cat allergies. The integration of anti‐Fel d 1 IgY into cat food represents a practical and safe strategy, while the Fel‐CuMV vaccine and monoclonal antibodies provide long‐term solutions to reduce Fel d 1 allergenicity. At the same time, environmental measures and the use of proteases can be effective in reducing allergen exposure at the household level.

Future research should focus on optimizing these strategies, exploring synergies between dietary, immunological, and genetic approaches to develop integrated and personalized solutions for managing cat allergies. A multidisciplinary approach, combining molecular biology, veterinary medicine, and food technology, will be essential to address this complex public health issue.

## Author Contributions

**Simone Colosimo:** writing – original draft, conceptualization. **Cristiana Indolfi:** conceptualization, writing – original draft. **Vittoria Frattolillo:** writing – review and editing. **Gianluca Mondillo:** writing – review and editing. **Alessandra Perrotta:** data curation, resources. **Mariapia Masino:** methodology. **Michele Miraglia del Giudice:** supervision.

## Conflicts of Interest

The authors declare no conflicts of interest.

## Data Availability

The authors have nothing to report.
